# Advances in the study of B cells in renal ischemia-reperfusion injury

**DOI:** 10.3389/fimmu.2023.1216094

**Published:** 2023-11-01

**Authors:** Hongzhao Fan, Jia Liu, Jiajia Sun, Guiwen Feng, Jinfeng Li

**Affiliations:** ^1^ Kidney Transplantation Unit, The First Affiliated Hospital of Zhengzhou University, Zhengzhou, China; ^2^ Dietetics Teaching and Research Section, Henan Medical College, Xinzheng, China

**Keywords:** renal ischemia-reperfusion injury, immune cells, B cells, Breg, B cell targeting

## Abstract

Renal ischemia-reperfusion injury (IRI) is a non-negligible clinical challenge for clinicians in surgeries such as renal transplantation. Functional loss of renal tubular epithelial cell (TEC) in IRI leads to the development of acute kidney injury, delayed graft function (DGF), and allograft rejection. The available evidence indicates that cellular oxidative stress, cell death, microvascular dysfunction, and immune response play an important role in the pathogenesis of IRI. A variety of immune cells, including macrophages and T cells, are actively involved in the progression of IRI in the immune response. The role of B cells in IRI has been relatively less studied, but there is a growing body of evidence for the involvement of B cells, which involve in the development of IRI through innate immune responses, adaptive immune responses, and negative immune regulation. Therefore, therapies targeting B cells may be a potential direction to mitigate IRI. In this review, we summarize the current state of research on the role of B cells in IRI, explore the potential effects of different B cell subsets in the pathogenesis of IRI, and discuss possible targets of B cells for therapeutic aim in renal IRI.

## 1 Introduction

Renal ischemia-reperfusion injury (IRI) is a common kidney disease that occurs as a result of kidney surgery, cardiac surgery, kidney transplantation, and disruption of the renal blood supply (1). During renal ischemia, renal tissues are affected by insufficient oxygen supply and accumulation of metabolites, leading to cellular damage and death. Then, the reperfusion process re-inflows blood into the kidney, but causes a series of inflammatory reactions and cellular damage that further aggravates kidney injury (2). The mechanisms of renal IRI include calcium overload, inflammatory response, reactive oxygen species damage and apoptosis, in which immune cell-mediated immune response also plays an important role (3–5). B lymphocytes are an important class of immune cells that usually produce antibodies, present antigens, and participate in immune regulation (6). More and more studies have shown that B lymphocytes play an important role in IRI. B lymphocytes are part of the inflammatory cells recruited to the kidney in renal IRI, involved in immune response exacerbates kidney damage. B cells participate in the immune response and inflammation by presenting antigens and secreting cytokines to cause further renal damage. And some B-cell subsets are immunosuppressive and play a protective role in IRI (7). In this review, we summarize the possible mechanisms of the role of B cells in renal IRI, including innate immune response, adaptive immune response, and negative immune regulation. This may help to provide new directions for the prevention and treatment of renal IRI in the future.

## 2 Molecular pathophysiology of IRI

Renal IRI is one of the main causes of acute kidney injury (AKI), which can lead to acute damage to renal tissue. Studies have shown a bidirectional interaction between AKI and the immune system. AKI provokes intrarenal, systemic inflammation and both innate and adaptive immune system that play important roles in the pathogenesis of kidney injury. B cells are related to AKI as part of innate and adaptive immunity (8). IRI involves both ischemic and reperfusion processes, ischemia leads to intracellular hypoxia, which promote the production of reactive oxygen species (ROS) and lead to tissue damage (9). In addition, cellular depletion of ATP allows sodium and water to enter the cell, causing significant cellular edema (10). These processes can cause disruption of the cytoskeleton, cell membrane and mitochondrial membrane (11).

During reperfusion, the accumulation of ROS aggravates cellular damage and the increasing intracellular calcium ion levels activate calpain (12). It also leads to mitochondrial dysfunction, causing the release of cytochrome C, mitochondrial DNA (mtDNA) and other substances (13). These can cause the release of multiple inflammatory cytokines that recruit immune cells to the injured tissue. Endothelial cells also produce vasoactive substances such as Platelet derived growth factor (PDGF), leading to vasoconstriction (14). Ultimately, tubular cells and vascular endothelium undergo damage due to this complex pro-inflammatory cascade response. In addition, elevated endothelin and thromboxane, together with decreased prostacyclin, can also lead to vasoconstriction and further aggravate tissue damage (15). IRI is accompanied by aseptic inflammation in which both the innate and adaptive immune systems are involved. Activation of the immune system will occur through damage-associated molecular pattern (DAMP) binding to toll-like receptor (TLR) and activation of the complement system, leading to further damage to renal tissue, increased tissue immunogenicity as well as initiation of fibrosis and thus conversion to chronic kidney diseae (CKD) (16).

Renal IRI is inevitable during renal transplantation and remains a key factor affecting the survival of the transplanted kidney, which may lead to DGF and primary renal graft nonfunction (PNF) (17). Meanwhile, a large amount of clinical evidence shows that the severity of IRI is positively correlated with complications such as rejection and the incidence of transplanted kidney failure (18, 19).

## 3 B cell associated immune response pattern of IRI

Immune cells are involved in the immune response of the body and protect the health of the body by exerting immune effects under physiological conditions (20), but can exacerbate tissue injury under certain pathological conditions (21). In renal IRI, The immune response is activated to mediate kidney and distant organ damage (22). In this process, various groups of immune cells interact with each other to form an interaction network. Among them, B cells not only directly mediate immune injury and chemotaxis of other immune cells enriched in inflammation, but also act as antigen-presenting cells to activate adaptive immunity (23, 24). B cells and other immune cells can also migrate to distant organs through circulating blood flow and other pathways, and through the secretion of cytokines and other active substances to affect distant organs, further leading to damage to distant organs, thereby increasing the burden on the body (25).

**Figure 1:**
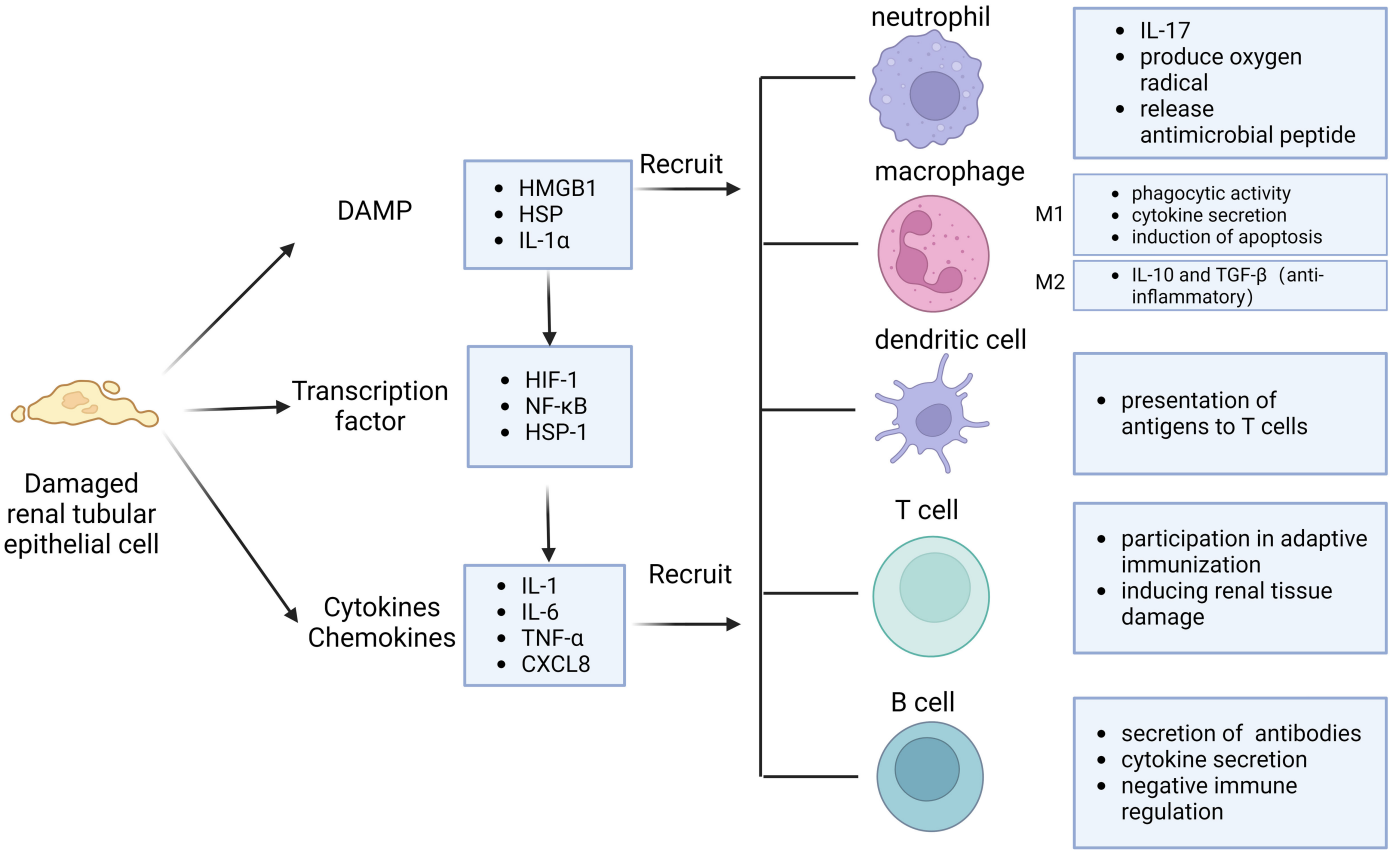
Immune response pattern of IRI. Injured renal tubular epithelial cells release damage-associated molecular pattern (DAMP) and cytokines, activate transcription factors. They can interact and recruit various immune cells, including neutrophils, macrophages, dendritic cells, T cells and B cells.

Potential mechanisms of immune cell activation and recruitment in the kidney after IRI involve DAMP (**Figure 1**). DAMP is an endogenous molecule expressed in cells under physiological conditions and released to the outside of the cell after tissue injury, which can effectively activate the immune system to initiate and maintain inflammatory responses by binding to pattern recognition receptors (PRR). DAMP molecules include high mobility group box 1 (HMGB1), histones, cell free DNA, IL-33, extracellular cold-inducible RNA-binding protein (eCIRP), and heat shock protein (HSP) (26). These histones released from necrotic tubules or DAMP such as HMGB1 activate TLRs on dendritic cells or macrophages and inflammatory vesicles in the cytoplasm to trigger the secretion of proinflammatory cytokines and chemokines in the ischemic kidney. TLR2 and TLR4 are similarly expressed on normal tubular epithelial cells and their expression is further increased after IRI (27). As part of intrinsic immunity, TLR4 plays an important role (28). Bergler et al. found that TLR4 was highly upregulated after renal IRI in a rat allogeneic kidney transplantation model and that high TLR4 expression was strongly associated with graft dysfunction (29). In addition, TLR4-deficient mice are protected from renal IRI and kidneys from donors with loss of TLR4 alleles exhibit fewer pro-inflammatory cytokines in the post-transplant kidney. Meanwhile, the onset of activation of intrarenal HIFs after IRI is upregulated mainly in renal tubular, mesenchymal and endothelial cells. Activation of intrarenal transcription factors such as nuclear factor κB (NF-κB), heat shock factor protein 1, and HIF-1α similarly stimulates the synthesis of a range of proinflammatory cytokines, such as IL-1, IL-6, and tumor necrosis factor (TNF) (30). Cytokines and chemokines are important mediators that regulate the recruitment of immune cells to the post-ischemic kidney, and they direct neutrophils and macrophages to the site of injury (31).

Neutrophils are the first immune cells to be recruited to the site of injury. Neutrophils may be involved in inducing renal injury by blocking renal microvessels and secreting oxygen free radicals and proteases. Neutrophils can produce oxygen radicals, release degranulating substances, platelet activating factors and other substances that damage kidney tissue, and can also release cytokines to recruit more neutrophils to form positive feedback, further aggravating tissue damage (32). Although resident macrophages are rare in normal kidneys, their numbers increase significantly in postischemic kidneys. IRI promotes endothelial damage and modification of heparin sulfate proteoglycans in microvascular basement membranes, facilitates their binding to L-selectin, and induces monocyte chemotactic protein 1 (MCP-1). These changes induce an early influx of monocytes and macrophages into the ischemic kidney. Monocytes adhere to blood vessels, and after IRI occurs, monocytes enter the tissue to form macrophages in response to the chemotaxis of damaged cells, inflammatory factors, and their neutrophils. Soon after kidney injury, macrophages become numerically dominant infiltrating cells (33). Macrophages can be divided into M1 and M2 according to their mode of action (34). M1, also known as classically activated macrophages, are activated to exert potent phagocytic activity and release several important cytokines, such as IL-1, IL-6, IL-8, IL-12, and TNF, as well as recruit neutrophils and induce apoptosis to promote the inflammatory cascade, thereby contributing to kidney injury (35). Therefore, inhibition of macrophage infiltration attenuated renal injury in a mouse renal IRI model (36). M2 product IL-10 and TGF-β, which are anti-inflammatory and moderate the immune response to tissue damage.

Many studies have revealed the important role of different lymphocyte subsets in IRI. The basic function of dendritic cells is to present antigens to T cells and act as messengers between the innate and adaptive immune systems. The binding of dendritic cells to the endothelium and their migration seems to be facilitated in the initial inflammatory response after IRI, leading to an increased ratio of myeloid to plasmacytoid dendritic cells, which can lead to delayed graft function and acute rejection. It has been found that T cells, especially CD4+ T cells, directly or indirectly promote the establishment of early kidney injury in IRI. T cell-targeting drugs, such as tacrolimus and mycophenolate mofetil, significantly attenuated early kidney injury after IRI. CD4, CD8 double-knockout mice were largely protected from early kidney injury, and the adhesion of their T cells to the tubular epithelial cells *in vitro* after hypoxia and reoxygenation was increased two-fold. Periplasmic transfer of T cells into these mice restored renal injury after IRI, suggesting that T cell deficiency enhances the protective effect of IRI on the kidney. The role of T cells appears to extend to the late stages of injury or repair of IRI and is not limited to the early stages of injury. Treg play a role in renal regeneration or nephroprotection.Treg promote tubular proliferation, which accelerates the repair process in the late and early recovery stages of injury after IRI (37, 38).

## 4 B cells in the IRI process

**Figure 2:**
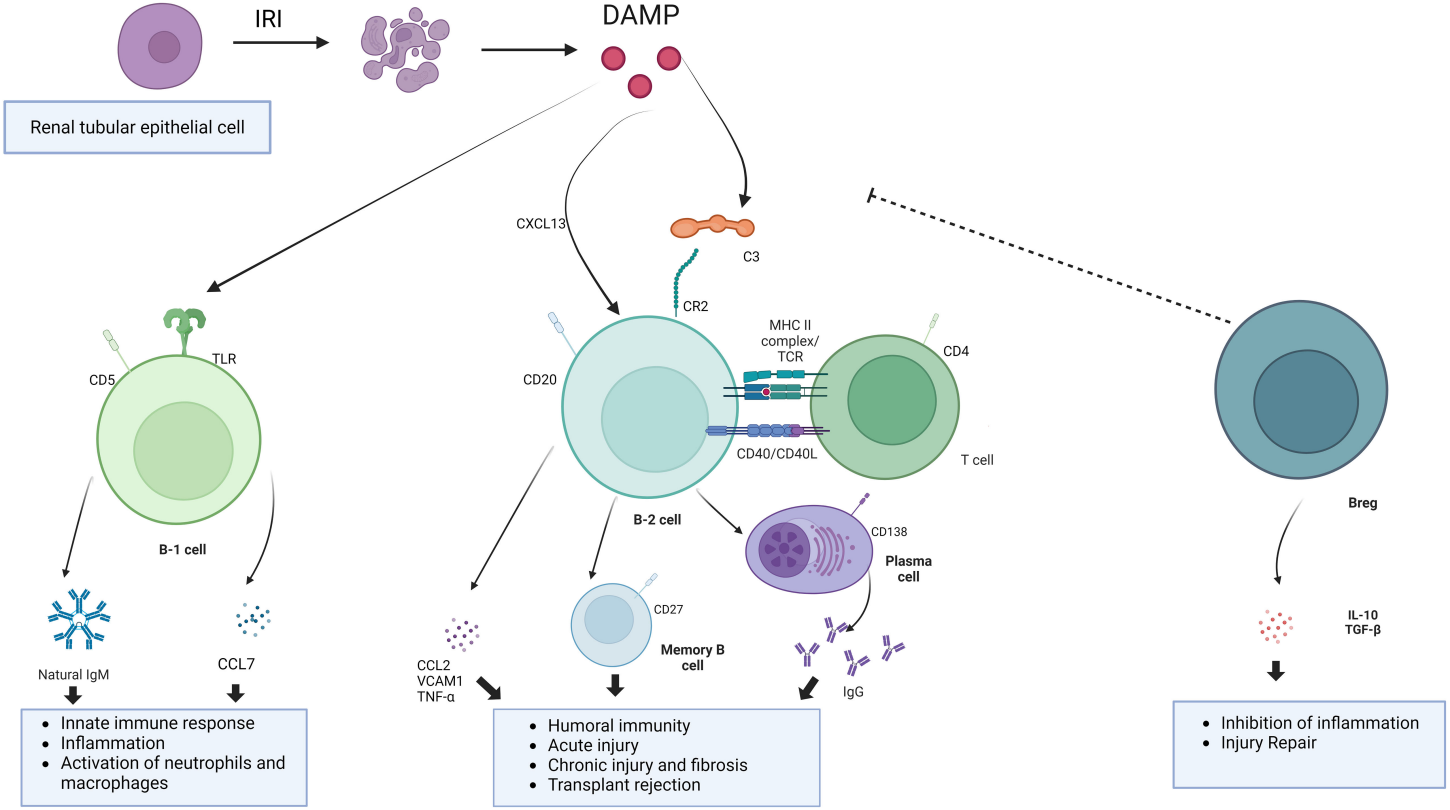
The mechanism of action of B cells. B cells involve in the development of IRI through innate immune responses, adaptive immune responses, and negative immune regulation.Undergoing renal IRI, injured renal tubular epithelial cells release DAMP, binding to toll-like receptors and activation of the complement system. B1 cells, as part of innate immunity, are activated to release natural antibodies and cytokines to aggravate the IRI. Damaged renal tubular epithelial cells could secrete Chemokines such as CXCL13 to recruit B cell. B cells can be activated by complement and T cells. Binding of C3dg to the B-cell receptor CR2 lowers the threshold for B-cell activation and promotes antibody production. Breg cells are immunosuppressive cells that support immunological tolerance.

IRI, as a aseptic inflammatory process, is involved in the activation of both innate and adaptive immunity. In previous studies, activated B cells were observed to be enriched in the kidney after IRI. These activated B cells can be divided into B1 cells and B2 cells according to the intrinsic or adaptive immune function they perform (39). Among them, B1 cells are intrinsic immune cells, accounting for about 5% to 10% of the total B cells, and play an important role in the early stages of the immune response. B1 cells can be further subdivided into subpopulations B1a and B1b based on the expression of the surface marker CD5. B1 cells comprise the majority of neonatal B cells and are derived primarily from fetal liver and omentum. In adults, B1 cells are found mainly in the pleural and peritoneal cavities and respond to T cell-dependent antigens by secreting polyreactive IgM antibodies (40). B2 cells, on the other hand, are the main cells that secrete antibodies to participate in the humoral immune response, appear relatively late in individual development, are localized in the follicular area of peripheral lymphoid organs (41). After maturation in the bone marrow, B cells migrate to lymphoid organs. B cells are activated in the spleen or other secondary lymphoid organs by contact with antigen via BCR. There are very few B cells in the normal kidney. In renal IRI, due to chemokine effects, activated B lymphocytes accumulate in the renal tissue (42). These activated B lymphocytes may be involved in the inflammatory response and immune response through the release of cytokines and chemokines (43). Also, B lymphocytes play a role in the pathological process by producing antibodies to mediate immune injury (44). These antibodies may activate adaptive immunity and trigger an inflammatory response by binding antigens on kidney tissue. At the same time, this process can exacerbate IRI and post-IRI rejection and chronic fibrosis (**Figure 2**).

### 4.1 Innate immune response

Natural and autoantibodies play a key role in the initiation of IRI. Ischemic tissues are exposed to certain autoantigens such as membrane linked protein IV, phospholipids, DNA, and histones, thus allowing certain pathogenic natural and autoantibodies to bind to ischemic tissue cells and activate the complement system, causing tissue injury (43). This also suggests that early plasma replacement in renal IRI may have a therapeutic role. B cells can express TLR, which recognize specific molecular patterns associated with injury and thus activate the immune response.

In 2003, Burne-Taney et al. found that after experiencing IRI, B cell-deficient mice had significantly less kidney injury and a much lower mortality rate compared to wild-type mice. This was the first confirmation that B cells play an important role in renal IRI. The authors searched for possible mechanisms of the role of B cells and showed that B cells may aggravate tissue injury by producing cytokines (45). However, specific serum factor types were not elaborated at the time. Subsequently, Renner et al. found an increase in natural antibody IgM in the mouse kidney tract after experiencing renal IRI, and depletion of B1 cells by intraperitoneal injection of distilled water before experiencing IRI successfully reduced tract IgM levels and could reduce renal injury to some extent (46). These results demonstrated that B1 cells acted on the aggravation process of renal IRI injury through the secretion of IgM. Inaba et al. found in a mouse model of renal IRI that the number of B cells in the spleen decreased and the number of B cells in the renal parenchyma increased within a few hours after injury, suggesting that B cells may be mobilized from the spleen and immunologically infiltrated into the kidney during renal IRI (47). They also found that after IRI, the B1 cell subset expressing CD5 molecules accounted for about 20% of the total number of cells in the kidney and was the most significantly increased B cell subset, while this B1 cell subset was almost undetectable in normal kidneys without IRI. At the same time, they hypothesized that high expression of CD11b on B1 cells is responsible for driving the enrichment of B cells into the kidneys. This confirms the role of B1 cells in renal IRI. Further studies found that B cells influence the recruitment of other immune cells to the kidneys by secreting chemokines CCL7. The transcriptome showed lower levels of CCL7, a chemokine that recruits neutrophils and monocytes to injured tissues, in the B cell-deficient mouse group after IRI, and revealed that B1a cells act on neutrophils and mononuclear macrophages by secreting CCL7 to cause infiltrative damage to the kidney (48, 49). And in human kidney biopsy specimens, CCL7 transcript levels were significantly higher in AKI kidneys than in kidneys with normal renal function, further demonstrating that B cells and CCL7 levels correlate with the degree of acute kidney injury (47). In contrast, in intestinal IRI, natural antibodies produced by B cells bind to neoantigens expressed in the intestine after I/R and cause tissue inflammation by activating the classical and lectin pathways of complement (50, 51). In summary, B cells participate in the innate immune response process of IRI through the production of natural immune antibody IgM and cytokine CCL7, which aggravate kidney injury (**Table 1**).

**Table 1:** phenotype and function of B1a.

Cell type	Phenotype	Function
B1a	CD5	B1a surface marker that regulates internal activation
	CD11b	mediating B cell migration
	IgM	exacerbation of early kidney injury by early humoral immune response
	CCL7	recruiting neutrophils and monocytes to injured tissues

### 4.2 Adaptive immunity

B cells undergo several stages in the bone marrow, including pro-B cells, pre-B cells, and immature B cells, and then differentiate into naïve B cells. Naïve B cells express both mIgM and mIgD on their surface. Naïve B cells leave the bone marrow and settle in the peripheral immune organs. Naïve B cells express CD19, CD20, etc. Activation of naïve B cells requires a first signal provided by specific antigens and a second signal provided by co-stimulatory molecules. The BCR complex consisting of CD19/CD21/CD81 on the surface of B cells greatly enhances the first signal of B cell activation. Activated B cells form primary focus at the junction of the T and B cell zones of peripheral lymphoid organs. B cells may differentiate directly into plasmoblasts secreting antibodies in primary focus, or they may migrate to lymphoid follicles to form germinal centers and undergo somatic high-frequency mutations, Ig affinity maturation, and class switching to differentiate into plasma cells or memory cells. The majority of memory B cells express CD27. Plasma cells do not express some B-cell surface markers and show some new plasma cell-specific markers, such as plasma cell antigen-1 (PCA-1), and highly express CD38 and CD138. B cells are involved in the immune response to IRI through antibody production, immunomodulation and intercellular interactions in adaptive immunity. Their functions play an important role in regulating the inflammatory response, promoting tissue repair, and maintaining immune homeostasis.

#### 4.2.1 B-cell recruitment in the IRI kidney

Kreimann et al. found that leukocyte infiltration increased from day 1 to day 7 of IRI. On day 7 after IRI, CD22+ B-cell infiltration was detected in ischemic kidneys, possibly due to CXCL13 recruitment. It was found that experiencing IRI could promote elevated CXCL13 levels and that elevated CXCL13 levels were positively correlated with the duration of IRI. Elevated serum CXCL13 and elevated levels of pro-inflammatory factors MCP1 and IL6 occurred at 24 hours of reperfusion, suggesting that IRI kidneys may begin recruiting B cells at an early stage. Sequencing analysis of IRI-injured kidneys revealed a population of CXCR5+ cells of B-cell origin in the kidney after experiencing IRI. Next, they performed kidney transplantation using 30 and 60 minutes of cold ischemia time and found that allogeneic kidney transplantation and longer cold ischemia time resulted in higher levels of CXCL13 and B-cell infiltration. Although CXCL13 is mainly secreted by follicular helper T (Tfh), some other cells such as damaged renal tubular epithelial cells may also be its source (52). In another lung IRI study, it was found that stromal cells secreting IL23 and CXCL12 were also able to promote B-cell recruitment after IRI (53).

#### 4.2.2 B cells are involved in IRI chronic injury

**Figure 3:**
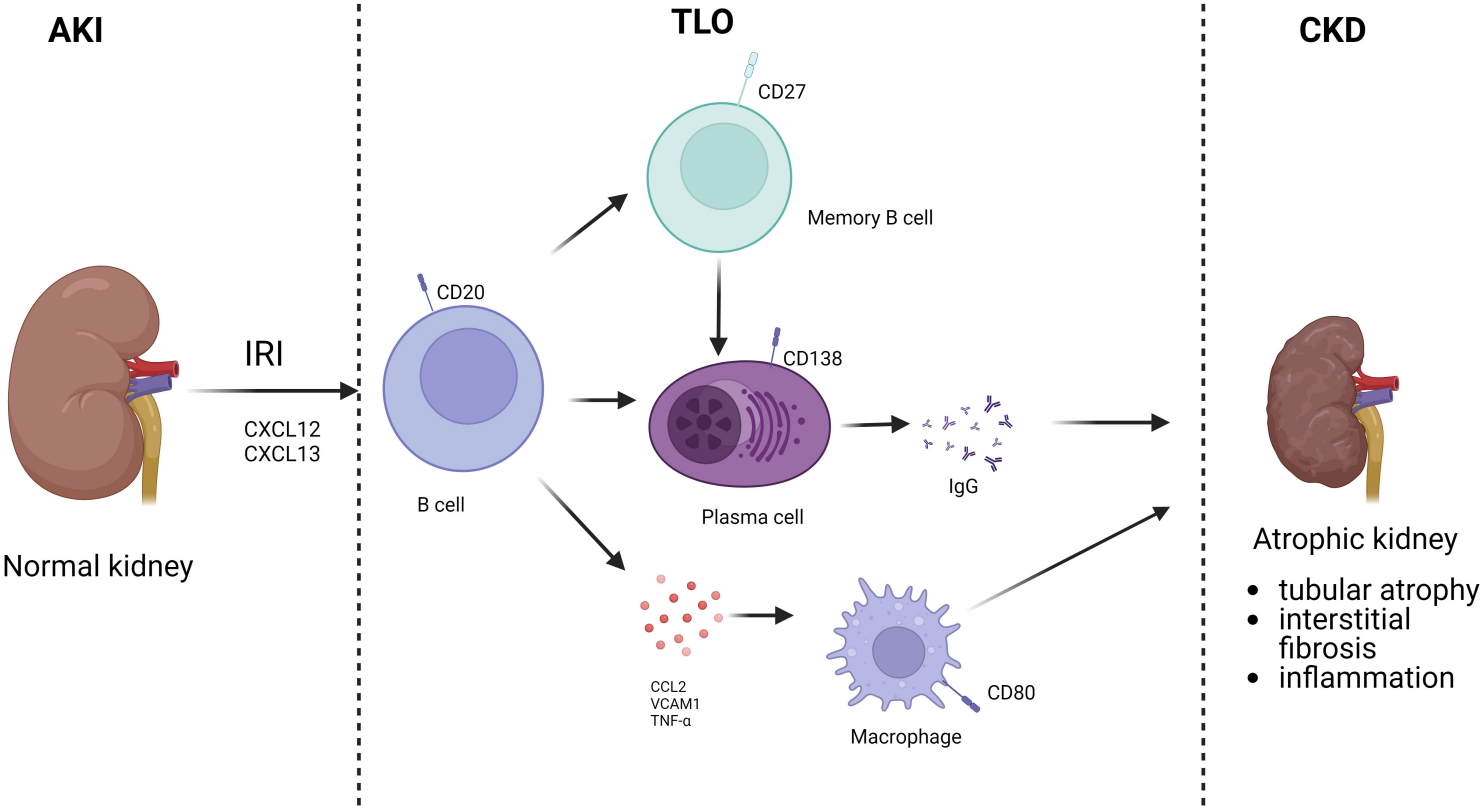
B cells involved in AKI to CKD. After AKI, B cells were recruited to the kidney. Activated B cells can differentiate into memory B cells and plasma cells, which secrete antibodies, and also directly secrete chemokines. Finally, TLO is formed locally in the kidney, which in turn leads to CKD.

B cells are involved not only in the acute response to ischemic injury through adaptive immunity but are also associated with repair after IRI. During IRI, the exposure of self-antigen leads to the activation of some B cells and their differentiation into memory B cells. When exposed to the same antigen again, memory B cells are rapidly activated and differentiated into plasma cells, which produce a large number of antibodies causing chronic kidney injury and rejection (54). Cippà showed that late B cell activity in renal allografts is closely associated with repair of dysfunctional kidneys.They simulated the transition of transplanted kidneys to CKD after undergoing bilateral renal ischemia-reperfusion through a mouse model, suggesting that B lymphocyte action is inherent in the late phase transition from acute kidney injury to CKD. This process was observed with a B-cell response similar to that of allograft biopsies from kidney transplant patients, initiating an antigen-driven immune process in the absence of foreign antigens (55). At 6 months after a single IRI, lymphocytes were the most abundant immune cells in the kidney, organized mainly in clusters of resolved renal tubular injury and into highly vascularized ectopic lymphoid structures populated by CD19 B and CD3 T cells, suggesting a greater recruitment of B cells in the later stages of IRI injury (56). B cell areas with CD19/CD45R germinal centers or B cell fractions dimly embedded in the CD21/CXCL13 follicular dendritic cell network were usually observed only in mature germinal centers separated from the highly proliferating Ki67 lymphocyte clusters. And systematic analysis of transcribed cytokines verified that this ectopic lymphoid tissue formation containing B cells was driven from acute to chronic inflammation by cytokines. Thus, in the absence of foreign antigens, T and B lymphocytes accumulate in the mouse kidney, transforming from acute to chronic injury. B lymphocytes are rare in normal kidneys, and transcriptional analysis showed a massive expansion of B lymphocytes in the following weeks after IRI, gradually switching to the plasma cell type. CXCL12, a cytokine highly expressed by the renal stroma during the transition to chronic injury. RNA sequencing profiles of IgM, IgG2c and IgA-like immunoglobulin gene transcripts and a significant increase in Ig kappa light chain were consistent with a significant increase in renal local production of antibodies is consistent. Thus, antibody-secreting cells accumulate in the kidney with the transition from acute to CKD. Analysis confirmed the presence of polyclonal B-cell populations in the kidney enriched for a limited number of dominant clones (57). Both histologic findings and BCR analysis provided evidence for proliferation, selection and maturation of B lymphocytes in the nephron’s germinal centers and the transition from acute kidney injury to CKD. In the absence of foreign antigens, intrarenal B-cell responses lead to the production of broadly reactive autoantibodies. Due to the high frequency of foreign major histocompatibility complex (MHC) molecules in B-cell precursors, donor antigens may contribute substantially and accelerate progression to this immune process in the context of transplantation (58). Liu et al. established a renal bilateral IRI recovery model to explore the transition from acute to chronic kidney injury in mice and found tubular atrophy, interstitial fibrosis, and inflammation to be the primary long-term outcomes of IRI. RNA-seq analysis identified a series of time-specific gene expression patterns associated with innate and adaptive immunity (56). Jang et al. found that in the absence of alloantigenic stimulation, B cells infiltrate the postischemic kidney and regulate the repair process of tubular cells after renal IRI. B cells in which can influence tubular atrophy and regeneration, while blocking CD126 improves repair after IRI. These data suggest that undergoing renal IRI can lead to B cell transport to the kidney and alter the tubular repair process. Furthermore, targeting CD126 represents a novel approach to improve clinical outcomes in renal IRI (22). In another study, Han et al. identified a possible mechanism by which B cells affect tubular repair: by establishing a model of unilateral ureteral obstruction (UUO)-induced tubular interstitial fibrosis, it was found that the kidneys of B cell-deficient μMT mice and anti-CD20-treated mice showed lower levels of mononuclear macrophage infiltration and collagen deposition compared to wild-type mice, and in both mouse models Levels of tumor necrosis factor alpha (TNF-α), vascular cell adhesion molecule 1 (VCAM-1), and CCL2 were decreased, suggesting that B cells may influence renal tubular epithelial cell repair after IRI by recruiting mononuclear macrophages through secretion of these substances (59). Renal IRI is inevitable during renal transplantation, while substantial clinical evidence suggests that the severity of IRI is positively correlated with complications such as rejection and the incidence of transplanted kidney failure. Einecke evaluated the significance of B-cell and plasma cell infiltration in renal allografts and found that B-cell-associated transcripts (BATs) and immune globulin transcripts (IGTs) scores were associated with tubular atrophy, interstitial inflammation and fibrosis (60). The presence of tertiary lymphoid organs (TLO) in grafts suggests that B cells may contribute to fibrosis (**Figure 3**). The study found a reduction in TLO formation by treating mice with aCD20, revealing that B cells are required for TLO formation in the graft model and that B cell depletion attenuates fibrosis, TLO formation and antibody secretion (53).

#### 4.2.3 T-B cell interactions

T cells are involved in renal IRI through adaptive immunity, and T cells play an important role in the injury process after IRI and in the transition from AKI to CKD (61, 62). T cells can be classified into Th1, Th2, Th17 and CD8-positive T cells according to their phenotype and function, and these different subtypes of T cells can participate in renal IRI by secreting cytokines, etc (63). T cells can also interact with B cells to participate in renal IRI. CD4 T cells were first found to promote antibody production by B cells, and Th1 and Th2 cells can promote B cell class switching and exert effects. In contrast, a unique subpopulation of T cells, Tfh, plays a key role in regulating T cell-dependent B cell responses. Tfh cells upregulate CXCR5, which allows them to homing to the interface between the T and B cell regions of the lymph node, where they interact with newly activated B cells through antigen presentation. B cells signal to Tfh cells via ICOS and IL-6, while Tfh cells provide CD40L and IL-21 to B cells. Through continuous interaction with B cells, Tfh cells promote survival, proliferation, Ig type switching, maturation and differentiation of B cells to memory B cells and antibody-producing plasma cells through germinal center responses (64, 65).

#### 4.2.4 Complement promotes B-cell activation

The complement system is an important component of the immune system, which is responsible for anti-infective, anti-tumor and immunomodulatory roles in the immunity of the body (66). Each complement component is relatively stable in the serum, with complement C3 being the most abundant (67). The biological effects of complement include enhanced phagocytosis, enhanced chemotaxis of phagocytes (68), can increase vascular permeability, has neutralizing viral, cytolytic effect, and modulation of immune response (69). Complement depletion impairs antibody production, and B cells express complement receptor 2 (CR2), which binds to C3dg, a C3 catabolic product that acts as a modulator. Antigen-specific B-cell receptors plus CR2-recognized C3dg encapsulated by antigen initiate phagocytosis and lower the threshold for B-cell activation, promoting antibody production (70). When complement receptors are absent on B cells, B cell activation is compromised. Also complement C3 can bind to CR2 on follicular DCs and promote B cell differentiation into mature plasma cells or memory B cells. The complement system can well link innate and adaptive immunity by delivering antigens to the B-cell compartment to enhance humoral immunity. Therefore, targeting complement to block B-cell activation and thus suppress humoral immunity is also a possible therapeutic modality (71, 72).

#### 42.5 B cells protected IRI through inhibiting immune response

Regulatory B cells are a subpopulation of B cells with immunosuppressive functions, accounting for about 10% of total B cells, which can exert regulatory functions by secreting negative cytokines such as IL-10 and transforming growth factor β (TGF-β) (73). They can also suppress self-reactive B cells and pathogenic T cells in a cell contact-dependent manner by expressing suppressor molecules on the cell surface (74), so Breg may play a negative regulatory role in IRI. Deng et al. found that some B-cell subsets can reduce renal IRI in mice by secreting IL-10 (75). Brandon et al. found that mice completely lacking mature B cells suffered more severe injury than wild-type mice due to a reduction in the number of IL-10-producing B cells during reperfusion, thus providing further evidence that in renal IRI The presence of a subpopulation of B cells that produce negative regulatory factors could protect the kidney from mitigating injury. Fang et al. infused Bregs into mice for one day before experiencing IRI, and after 2 days compared to the non-infused Breg group could significantly reduce blood creatinine, urea nitrogen and the degree of tubular injury. the level of renal B cell infiltration in the infused Breg group decreased, while the Breg and Treg renal infiltration levels were significantly increased. The study then constructed an IRI model for 1 day after infusion of Bregs,mice were harvested 3 days after IRI, and found that infusion of Breg could also reduce renal injury in this model. Thus, Breg can both reduce acute kidney injury after IRI and promote kidney repair after IRI. Immediately prior to IRI the authors treated with both anti-CD45RB combined with or without anti-Tim-1 antibodies and found that both attenuated the extent of renal tubular injury by promoting IL-10 Bregs production (76). Royster et al. found that in mouse intestinal IRI, B1a cells not only produced IgM to provide natural immune aggravation of injury, but also produced IL10 inflammatory mediators to regulate immunity and suppress inflammatory responses, and by injecting B1a cells from the peritoneal cavity could attenuate serum levels of organ damage markers such as ALT and AST and the inflammatory factor IL6 (50). while in brain IRI, infarct volume and injury after middle cerebral artery occlusion (MCAO) could be attenuated by transferring Breg (77).

## 5 Mitigating renal IRI from a B-cell therapy perspective

Given the important role played by B-cell kidney IRI process, targeting B cells and antibodies or increasing Breg may be effective in reducing IRI. Current clinical therapeutic approaches that target B cells include killing B cells directly, modulating B cell function, and inhibiting molecules that are dependent on B cell survival (78). According to the different mechanisms of action, drugs acting on B cells are classified as anti-CD20(CD20 expressed in B cells at all developmental stages except plasma cells and can affect the proliferation and differentiation of B cells by regulating the transmembrane flow of calciumions (79).) class drugs, anti-CD19 (CD19 is a key signaling molecule in the process of antigen recognition by B-cell surface receptors) class drugs, anti-B cell activating factor [BAFF, a crucial factor for B cell survival, binds to the B cell surface receptor to support B cell survival, proliferation and differentiation (80)] and Bruton’s tyrosine kinase [BTK, a key kinase in the BCR signaling pathway that is involved in B-cell proliferation, differentiation and apoptosis (81, 82)] inhibitors (83). However, these drugs and methods of killing B cells or inhibiting B cell function carry a significant risk of immunocompromise as well as pathogen infection, and their use in the prevention and treatment of renal IRI is generally considered to be more costly than it is worth. Moreover, nonspecific removal of B cells suppresses the immunoregulatory function of Breg cells, which is very detrimental to overall immune homeostasis.

Plasma replacement is the non-selective removal of macromolecules, such as antibodies, complement, immune complexes and coagulation factors, from plasma by physical means. The use of melphalan and plasma exchange to prevent antibody-mediated rejection in renal transplantation has been advanced only in pre-sensitized patients, and we do not know their role in the prevention of renal IRI (84). Given the negative effects of B1 cells in the early stages of renal IRI, blocking B1 cell function is a potential direction worth considering. The mouse data suggest that CCL7 blockade may be a useful therapeutic strategy to reduce the infiltration of inflammatory cells into the kidneys, thereby improving AKI, without affecting the systemic mobilization of bone marrow cells, which may contribute to the defense against infection (47). Because B cells are an important source of CCL7, observations in siglecg ^-/-^ mice suggest that targeting this cell subpopulation, particularly B1a cells, may be particularly useful. In this regard, siglecg itself may be a direct target: siglecg is a surface glycoprotein with an immunoreceptor tyrosine-based inhibition motif (ITIM) in its cytoplasmic structural domain that allows it to inhibit bcr-mediated activation (85). Therefore, the use of agonists (e.g., sialic acid) bound to siglecg may have therapeutic potential. One problem with inhibiting B1a function is that this subpopulation may also be an important source of the immunomodulatory cytokine IL-10 (86). Thus, inhibition of this subpopulation may be undesirable given the potential beneficial role of regulatory B cells in AKI models. In addition, the relatively small number of B1 cells in humans and the absence of specific markers also make translation to the clinic relatively difficult. CXCL13 serves as a major cytokine for B-cell chemotaxis, and the current study also established that CXCR5-positive B cells are enriched at sites of inflammation by CXCL13 released by injured renal tubular cells (87). Blocking CXCL13-CXCR5 may be effective in inhibiting B cell enrichment in the damaged kidney. In addition, CXCL13 release may also be regulated through TLR2 and IL-10 dependent mechanisms (88). Complement activation may also affect immune cell-mediated secretion of CXCL13 release. Thus, inhibition of CXCL13 may exert its protective effects against renal IRI through pathways other than B cells. It is important to note that CXCR5 expression was not limited to B cells; macrophages and Tfh cells were also identified as CXCR5-positive. Blocking CXCL13-CXCR5 may cause a decrease in the chemotaxis of these cells (52). Inhibition of specific T-cell activation and complement function has a positive role in renal IRI, but these studies lacked evidence of a B-cell pathway of action. Therefore, the primary place of direct blockade of T-B cell action and complement-B cell activation in protecting against renal IRI remains uncertain. By increasing the number of Breg and thus inhibiting the function of other immune cells and reducing the inflammatory response, exogenous infusion of Bregs may therefore be a new therapeutic route for the treatment of IRI. However, exogenous induction of B-cell differentiation to Breg is also a potential therapeutic modality and is simpler since *in vitro* isolation and culture of Breg takes longer time and carries more risk of contamination (89, 90). Fang et al. successfully induced Breg production using anti-CD45RB and produced the same therapeutic effect as exogenous direct infusion of Breg, both of which reduced renal IRI (76).

There are still no immune drugs specific for renal IRI, and there are only a few clinical trials targeting immune cells (91–93).

## 6 Conclusion and future prospects

In this review, we describe the mechanisms of renal IRI, the role played by immune cells, especially B cells, in IRI through multiple pathways, and explore potential therapeutic directions to mitigate IRI by targeting B cells and exogenous infusion of Breg. For renal IRI, we found that there is no single therapeutic approach that can fully address the renal effects of IRI, and therefore a combination of therapeutic approaches may be required. In this paper, we briefly describe some new therapeutic targets in terms of B cells, such as cytokine, T cell, plasma replacement and promotion of B cell differentiation to Breg. Although sufficient basic experimental and clinical data are needed before clinical practice, reducing renal IRI by acting on B cells is a promising research and therapeutic direction.

## Author contributions

HF, JL, JS and GF conceived the conception, analyzed data, and wrote the manuscript. JFL conceived the conception and performed critical revision of the manuscript. All authors read and approved the final manuscript. HF and JL contributed equally to this work.

## References

[1] Shiva N Sharma N Kulkarni YA Mulay SR Gaikwad AB. Renal ischemia/reperfusion injury: An insight on in *vitro* and in *vivo* models. Life Sci (2020) 256:117860. doi: 10.1016/j.lfs.2020.117860 32534037

[2] Zhuang S Xia S Huang P Wu J Qu J Chen R. Targeting P2RX1 alleviates renal ischemia/reperfusion injury by preserving mitochondrial dynamics. Pharmacol Res (2021) 170:105712. doi: 10.1016/j.phrs.2021.105712 34091010

[3] Reid S Scholey JW. Recent approaches to targeting canonical NFκB signaling in the early inflammatory response to renal IRI. J Am Soc Nephrol (2021) 32(9):2117–24. doi: 10.1681/asn.2021010069 PMC8729839 34108233

[4] Yan JJ Ryu JH Piao H Hwang JH Han D Lee SK. Granulocyte colony-stimulating factor attenuates renal ischemia-reperfusion injury by inducing myeloid-derived suppressor cells. J Am Soc Nephrol (2020) 31(4):731–46. doi: 10.1681/asn.2019060601 PMC7191933 32132198

[5] Jeon J Lee K Yang KE Lee JE Kwon GAY Huh W. Dietary modification alters the intrarenal immunologic micromilieu and susceptibility to ischemic acute kidney injury. Front Immunol (2021) 12:621176. doi: 10.3389/fimmu.2021.621176 33777001 PMC7991094

[6] Wang H Morse HC 3rd, Bolland S. Transcriptional control of mature B cell fates. Trends Immunol (2020) 41(7):601–13. doi: 10.1016/j.it.2020.04.011 32446878

[7] Cyster JG Allen CDC. B cell responses: cell interaction dynamics and decisions. Cell (2019) 177(3):524–40. doi: 10.1016/j.cell.2019.03.016 PMC6538279 31002794

[8] Chen DQ Guo Y Li X Zhang GAQ Li P. Small molecules as modulators of regulated cell death against ischemia/reperfusion injury. Med Res Rev (2022) 42(6):2067–101. doi: 10.1002/med.21917 35730121

[9] Chen W Li D. Reactive oxygen species (ROS)-responsive nanomedicine for solving ischemia-reperfusion injury. Front Chem (2020) 8:732. doi: 10.3389/fchem.2020.00732 32974285 PMC7472733

[10] Zhao M Wang Y Li L Liu S Wang C Yuan Y. Mitochondrial ROS promote mitochondrial dysfunction and inflammation in ischemic acute kidney injury by disrupting TFAM-mediated mtDNA maintenance. Theranostics (2021) 11(4):1845–63. doi: 10.7150/thno.50905 PMC7778599 33408785

[11] Hayashida K Takegawa R Shoaib M Aoki T Choudhary RC Kuschner CE. Mitochondrial transplantation therapy for ischemia reperfusion injury: a systematic review of animal and human studies. J Transl Med (2021) 19(1):214. doi: 10.1186/s12967-021-02878-3 34001191 PMC8130169

[12] Pefanis A Ierino FL Murphy JM Cowan PJ. Regulated necrosis in kidney ischemia-reperfusion injury. Kidney Int (2019) 96(2):291–301. doi: 10.1016/j.kint.2019.02.009 31005270

[13] Liu X Murphy MP Xing W Wu H Zhang R Sun H. Mitochondria-targeted antioxidant MitoQ reduced renal damage caused by ischemia-reperfusion injury in rodent kidneys: Longitudinal observations of T(2) -weighted imaging and dynamic contrast-enhanced MRI. Magn Reson Med (2018) 79(3):1559–67. doi: 10.1002/mrm.26772 PMC5811825 28608403

[14] Chiba T Cerqueira DM Li Y Bodnar AJ Mukherjee E Pfister K. Endothelial-Derived miR-17∼92 Promotes Angiogenesis to Protect against Renal Ischemia-Reperfusion Injury. J Am Soc Nephrol (2021) 32(3):553–62. doi: 10.1681/asn.2020050717 PMC7920169 33514560

[15] Farzamfar S Hasanpour A Nazeri N Razavi H Salehi M Shafei S. Extracellular micro/nanovesicles rescue kidney from ischemia-reperfusion injury. J Cell Physiol (2019) 234(8):12290–300. doi: 10.1002/jcp.27998 PMC13484119 30609022

[16] Nieuwenhuijs-Moeke GJ Pischke SE Berger SP Sanders J SF Pol R A Struys M. Ischemia and reperfusion injury in kidney transplantation: relevant mechanisms in injury and repair. J Clin Med (2020) 9(1):253. doi: 10.3390/jcm9010253 31963521 PMC7019324

[17] Lindeman JH Wijermars LG Kostidis S Mayboroda OA Harms AC Hankemeier T. Results of an explorative clinical evaluation suggest immediate and persistent post-reperfusion metabolic paralysis drives kidney ischemia reperfusion injury. Kidney Int (2020) 98(6):1476–88. doi: 10.1016/j.kint.2020.07.026 32781105

[18] Hansson J Mjörnstedt L Lindnér P. The risk of graft loss 5 years after kidney transplantation is increased if cold ischemia time exceeds 14 hours. Clin Transplant (2018) 32(9):e13377. doi: 10.1111/ctr.13377 30098052

[19] Tingle SJ Figueiredo RS Moir JA Goodfellow M Talbot D Wilson CH. Machine perfusion preservation versus static cold storage for deceased donor kidney transplantation. Cochrane Database Syst Rev (2019) 3(3):Cd011671. doi: 10.1002/14651858.CD011671.pub2 30875082 PMC6419919

[20] Frasca D Blomberg BB Garcia D Keilich SR Haynes L. Age-related factors that affect B cell responses to vaccination in mice and humans. Immunol Rev (2020) 296(1):142–54. doi: 10.1111/imr.12864 PMC7371527 32484934

[21] Divangahi M Aaby P Khader SA Barreiro LB Bekkering S Chavakis. Trained immunity, tolerance, priming and differentiation: distinct immunological processes. Nat Immunol (2021) 22(1):2–6. doi: 10.1038/s41590-020-00845-6 33293712 PMC8020292

[22] Jang HR Gandolfo MT Ko GJ Satpute SR Racusen L Rabb H. B cells limit repair after ischemic acute kidney injury. J Am Soc Nephrol (2010) 21(4):654–65. doi: 10.1681/asn.2009020182 PMC2844308 20203156

[23] Karasawa K Asano K Moriyama S Ushiki M Monya M Iida M. Vascular-resident CD169-positive monocytes and macrophages control neutrophil accumulation in the kidney with ischemia-reperfusion injury. J Am Soc Nephrol (2015) 26(4):896–906. doi: 10.1681/asn.2014020195 25266072 PMC4378108

[24] Huang Q Niu Z Tan J Yang J Liu Y Ma H. IL-25 elicits innate lymphoid cells and multipotent progenitor type 2 cells that reduce renal ischemic/reperfusion injury. J Am Soc Nephrol (2015) 26(9):2199–211. doi: 10.1681/asn.2014050479 PMC4552110 25556172

[25] Ferhat M Robin A Giraud S Sena S Goujon J M Touchard G. Endogenous IL-33 contributes to kidney ischemia-reperfusion injury as an alarmin. J Am Soc Nephrol (2018) 29(4):1272–88. doi: 10.1681/asn.2017060650 PMC5875946 29436517

[26] Sosa RA Terry AQ Kaldas FM Jin YP Rossetti M Ito T. Disulfide high-mobility group box 1 drives ischemia-reperfusion injury in human liver transplantation. Hepatology (2021) 73(3):1158–75. doi: 10.1002/hep.31324 PMC8722704 32426849

[27] Gong T Liu L Jiang W Zhou R. DAMP-sensing receptors in sterile inflammation and inflammatory diseases. Nat Rev Immunol (2020) 20(2):95–112. doi: 10.1038/s41577-019-0215-7 31558839

[28] Wu H Chen G Wyburn KR Yin J Bertolino P Eris JM. TLR4 activation mediates kidney ischemia/reperfusion injury. J Clin Invest (2007) 117(10):2847–59. doi: 10.1172/jci31008 PMC1974864 17853945

[29] Hoffmann U Bergler T Rihm M Pace C Krüger B Jung B. Impact of Toll-like receptor 2 expression in renal allograft rejection. Nephrol Dial Transplant (2011) 26(3):1080–7. doi: 10.1093/ndt/gfq420 20628182

[30] Mcgettrick AF O'neill L. The role of HIF in immunity and inflammation. Cell Metab (2020) 32(4):524–36. doi: 10.1016/j.cmet.2020.08.002 32853548

[31] Jarczak D Nierhaus A. Cytokine storm-definition, causes, and implications. Int J Mol Sci (2022) 23(19):11740. doi: 10.3390/ijms231911740 36233040 PMC9570384

[32] He Y Li H Yao J Zhong H Kuang Y Li X. HO−1 knockdown upregulates the expression of VCAM−1 to induce neutrophil recruitment during renal ischemia−reperfusion injury. Int J Mol Med (2021) 48(4):185. doi: 10.3892/ijmm.2021.5018 34368855 PMC8416149

[33] Li L Huang L Vergis AL Ye H Bajwa A Narayan V. IL-17 produced by neutrophils regulates IFN-gamma-mediated neutrophil migration in mouse kidney ischemia-reperfusion injury. J Clin Invest (2010) 120(1):331–42. doi: 10.1172/jci38702 PMC2798679 20038794

[34] Yunna C Mengru H Lei W Weidong C. Macrophage M1/M2 polarization. Eur J Pharmacol (2020) 877:173090. doi: 10.1016/j.ejphar.2020.173090 32234529

[35] Ma S Wang DH. Knockout of trpa1 exacerbates renal ischemia-reperfusion injury with classical activation of macrophages. Am J Hypertens (2021) 34(1):110–6. doi: 10.1093/ajh/hpaa162 33005917

[36] Hasegawa S Inoue T Nakamura Y Fukaya D Uni R Wu CH. Activation of sympathetic signaling in macrophages blocks systemic inflammation and protects against renal ischemia-reperfusion injury. J Am Soc Nephrol (2021) 32(7):1599–615. doi: 10.1681/asn.2020121723 PMC8425643 33875568

[37] Dai H Thomson AW Rogers NM. Dendritic cells as sensors, mediators, and regulators of ischemic injury. Front Immunol (2019) 10:2418. doi: 10.3389/fimmu.2019.02418 31681306 PMC6803430

[38] Danelli L Madjene LC Madera-Salcedo I Gautier G Pacreau E Ben Mkaddem S. Early phase mast cell activation determines the chronic outcome of renal ischemia-reperfusion injury. J Immunol (2017) 198(6):2374–82. doi: 10.4049/jimmunol.1601282 28167630

[39] Eibel H Kraus H Sic H Kienzler AK Rizzi M. B cell biology: an overview. Curr Allergy Asthma Rep (2014) 14(5):434. doi: 10.1007/s11882-014-0434-8 24633618

[40] Vergani S Yuan J. Developmental changes in the rules for B cell selection. Immunol Rev (2021) 300(1):194–202. doi: 10.1111/imr.12949 33501672

[41] Shen P Fillatreau S. Antibody-independent functions of B cells: a focus on cytokines. Nat Rev Immunol (2015) 15(7):441–51. doi: 10.1038/nri3857 26065586

[42] Wang Y Liu J Burrows PD Wang JY. B cell development and maturation. Adv Exp Med Biol (2020) 1254:1–22. doi: 10.1007/978-981-15-3532-1_1 32323265

[43] Montecino-Rodriguez E Dorshkind K. B-1 B cell development in the fetus and adult. Immunity (2012) 36(1):13–21. doi: 10.1016/j.immuni.2011.11.017 22284417 PMC3269035

[44] Zhang Y Garcia-Ibanez L Toellner KM. Regulation of germinal center B-cell differentiation. Immunol Rev (2016) 270(1):8–19. doi: 10.1111/imr.12396 26864101 PMC4755139

[45] Burne-Taney MJ Ascon DB Daniels F Racusen L Baldwin W Rabb H. B cell deficiency confers protection from renal ischemia reperfusion injury. J Immunol (2003) 171(6):3210–5. doi: 10.4049/jimmunol.171.6.3210 12960350

[46] Renner B Strassheim D Amura CR Kulik L Ljubanovic D Glogowska MJ. B cell subsets contribute to renal injury and renal protection after ischemia/reperfusion. J Immunol (2010) 185(7):4393–400. doi: 10.4049/jimmunol.0903239 PMC3133676 20810984

[47] Inaba A Tuong ZK Riding AM Mathews R J Martin J L. B lymphocyte-derived CCL7 augments neutrophil and monocyte recruitment, exacerbating acute kidney injury. J Immunol (2020) 205(5):1376–84. doi: 10.4049/jimmunol.2000454 PMC7444279 32737150

[48] Mercer PF Williams AE Scotton CJ José RJ Sulikowski M Moffatt JD. Proteinase-activated receptor-1, CCL2, and CCL7 regulate acute neutrophilic lung inflammation. Am J Respir Cell Mol Biol (2014) 50(1):144–57. doi: 10.1165/rcmb.2013-0142OC PMC3930934 23972264

[49] Struyf S Gouwy M Dillen C Proost P Opdenakker G Van Damme J. Chemokines synergize in the recruitment of circulating neutrophils into inflamed tissue. Eur J Immunol (2005) 35(5):1583–91. doi: 10.1002/eji.200425753 15827963

[50] Royster W Ochani M Aziz M Wang P. Therapeutic potential of B-1a cells in intestinal ischemia-reperfusion injury. J Surg Res (2021) 268:326–36. doi: 10.1016/j.jss.2021.06.070 PMC8678159 34399355

[51] Zhang M Alicot EM Carroll MC. Human natural IgM can induce ischemia/reperfusion injury in a murine intestinal model. Mol Immunol (2008) 45(15):4036–9. doi: 10.1016/j.molimm.2008.06.013 PMC3230121 18672288

[52] Kreimann K Jang MS Rong S Greite R Von Vietinghoff S Schmitt R. Ischemia reperfusion injury triggers CXCL13 release and B-cell recruitment after allogenic kidney transplantation. Front Immunol (2020) 11:1204. doi: 10.3389/fimmu.2020.01204 32849490 PMC7424013

[53] Watanabe T Martinu T Chruscinski A Boonstra K Joe B Horie M. A B cell-dependent pathway drives chronic lung allograft rejection after ischemia-reperfusion injury in mice. Am J Transplant (2019) 19(12):3377–89. doi: 10.1111/ajt.15550 31365766

[54] Liu Y Hu J Liu D Zhou S Liao J Liao G. Single-cell analysis reveals immune landscape in kidneys of patients with chronic transplant rejection. Theranostics (2020) 10(19):8851–62. doi: 10.7150/thno.48201 PMC7392010 32754283

[55] Cornell LD Smith RN Colvin RB. Kidney transplantation: mechanisms of rejection and acceptance. Annu Rev Pathol (2008) 3:189–220. doi: 10.1146/annurev.pathmechdis.3.121806.151508 18039144

[56] Liu J Kumar S Dolzhenko E Alvarado GF Guo J Lu C. Molecular characterization of the transition from acute to chronic kidney injury following ischemia/reperfusion. JCI Insight (2017) 2(18):e94716. doi: 10.1172/jci.insight.94716 28931758 PMC5612583

[57] Arnaout R Lee W Cahill P Honan T Sparrow T Weiand M. High-resolution description of antibody heavy-chain repertoires in humans. PloS One (2011) 6(8):e22365. doi: 10.1371/journal.pone.0022365 21829618 PMC3150326

[58] Cippà PE Liu J Sun B Kumar S Naesens M Mcmahon AP. A late B lymphocyte action in dysfunctional tissue repair following kidney injury and transplantation. Nat Commun (2019) 10(1):1157. doi: 10.1038/s41467-019-09092-2 30858375 PMC6411919

[59] Han H Zhu J Wang Y Zhu Z Chen Y Lu L. Renal recruitment of B lymphocytes exacerbates tubulointerstitial fibrosis by promoting monocyte mobilization and infiltration after unilateral ureteral obstruction. J Pathol (2017) 241(1):80–90. doi: 10.1002/path.4831 27763657 PMC6680279

[60] Einecke G Reeve J Mengel M Sis B Bunnag S Mueller TF. Expression of B cell and immunoglobulin transcripts is a feature of inflammation in late allografts. Am J Transplant (2008) 8(7):1434–43. doi: 10.1111/j.1600-6143.2008.02232.x 18444922

[61] Göcze I Ehehalt K Zeman F Riquelme P Pfister K Graf BM. Postoperative cellular stress in the kidney is associated with an early systemic γδ T-cell immune cell response. Crit Care (2018) 22(1):168. doi: 10.1186/s13054-018-2094-x 29973233 PMC6030780

[62] Burne MJ Daniels F El Ghandour A Mauiyyedi S Colvin RB O'donnell MP. Identification of the CD4(+) T cell as a major pathogenic factor in ischemic acute renal failure. J Clin Invest (2001) 108(9):1283–90. doi: 10.1172/jci12080 PMC209434 11696572

[63] Satpute SR Park JM Jang HR Agreda P Liu M Gandolfo MT. The role for T cell repertoire/antigen-specific interactions in experimental kidney ischemia reperfusion injury. J Immunol (2009) 183(2):984–92. doi: 10.4049/jimmunol.0801928 19561110

[64] Kim MG Koo TY Yan JJ Lee E Han KH Jeong JC. IL-2/anti-IL-2 complex attenuates renal ischemia-reperfusion injury through expansion of regulatory T cells. J Am Soc Nephrol (2013) 24(10):1529–36. doi: 10.1681/asn.2012080784 PMC3785269 23833258

[65] Mehrotra P Patel JB Ivancic CM Collett JA Basile DP. Th-17 cell activation in response to high salt following acute kidney injury is associated with progressive fibrosis and attenuated by AT-1R antagonism. Kidney Int (2015) 88(4):776–84. doi: 10.1038/ki.2015.200 PMC4589446 26200947

[66] Pouw RB Ricklin D. Tipping the balance: intricate roles of the complement system in disease and therapy. Semin Immunopathol (2021) 43(6):757–71. doi: 10.1007/s00281-021-00892-7 PMC8547127 34698894

[67] Elieh Ali Komi D Shafaghat F Kovanen PT Kovanen PT Meri S. Mast cells and complement system: Ancient interactions between components of innate immunity. Allergy (2020) 75(11):2818–28. doi: 10.1111/all.14413 32446274

[68] Miyagawa S Maeda A Toyama C Kogata S Okamatsu C Yamamoto R. Aspects of the complement system in new era of xenotransplantation. Front Immunol (2022) 13:860165. doi: 10.3389/fimmu.2022.860165 35493484 PMC9046582

[69] Huber-Lang M Ekdahl KN Wiegner R Fromell K Nilsson B. Auxiliary activation of the complement system and its importance for the pathophysiology of clinical conditions. Semin Immunopathol (2018) 40(1):87–102. doi: 10.1007/s00281-017-0646-9 28900700 PMC5794838

[70] Fang Y Xu C Fu YX Holers VM Molina H. Expression of complement receptors 1 and 2 on follicular dendritic cells is necessary for the generation of a strong antigen-specific IgG response. J Immunol (1998) 160(11):5273–9. doi: 10.4049/jimmunol.160.11.5273 9605124

[71] Dempsey PW Allison ME Akkaraju S Goodnow CC Fearon DT. C3d of complement as a molecular adjuvant: bridging innate and acquired immunity. Science (1996) 271(5247):348–50. doi: 10.1126/science.271.5247.348 8553069

[72] Gonzalez SF Lukacs-Kornek V Kuligowski MP Pitcher LA Degn SE Turley SJ. Complement-dependent transport of antigen into B cell follicles. J Immunol (2010) 185(5):2659–64. doi: 10.4049/jimmunol.1000522 PMC3477863 20724732

[73] Rosser EC Mauri C. Regulatory B cells: origin, phenotype, and function. Immunity (2015) 42(4):607–12. doi: 10.1016/j.immuni.2015.04.005 25902480

[74] Catalán D Mansilla MA Ferrier A Soto L Oleinika K Aguillón JC. Immunosuppressive mechanisms of regulatory B cells. Front Immunol (2021) 12:611795. doi: 10.3389/fimmu.2021.611795 33995344 PMC8118522

[75] Deng J Kohda Y Chiao H Wang Y Hu X Hewitt SM. Interleukin-10 inhibits ischemic and cisplatin-induced acute renal injury. Kidney Int (2001) 60(6):2118–28. doi: 10.1046/j.1523-1755.2001.00043.x 11737586

[76] Fang T Koo TY Lee JG Jang JY Xu Y Hwang JH. Anti-CD45RB antibody therapy attenuates renal ischemia-reperfusion injury by inducing regulatory B cells. J Am Soc Nephrol (2019) 30(10):1870–85. doi: 10.1681/asn.2018101067 PMC6779371 31296607

[77] Bodhankar S Chen Y Vandenbark AA Murphy SJ Offner H. Treatment of experimental stroke with IL-10-producing B-cells reduces infarct size and peripheral and CNS inflammation in wild-type B-cell-sufficient mice. Metab Brain Dis (2014) 29(1):59–73. doi: 10.1007/s11011-013-9474-3 24374817 PMC3944055

[78] Zeiser R Sarantopoulos S Blazar BR. B-cell targeting in chronic graft-versus-host disease. Blood (2018) 131(13):1399–405. doi: 10.1182/blood-2017-11-784017 PMC6031308 29437591

[79] Kläsener K Jellusova J Andrieux G Salzer U Böhler C Steiner SN. CD20 as a gatekeeper of the resting state of human B cells. Proc Natl Acad Sci U.S.A. (2021) 118(7):e2021342118. doi: 10.1073/pnas.2021342118 33563755 PMC7896350

[80] Mackay F Browning JL. BAFF: a fundamental survival factor for B cells. Nat Rev Immunol (2002) 2(7):465–75. doi: 10.1038/nri844 12094221

[81] Sun C Nierman P Kendall EK Cheung J Gulrajani M Herman SEM. Clinical and biological implications of target occupancy in CLL treated with the BTK inhibitor acalabrutinib. Blood (2020) 136(1):93–105. doi: 10.1182/blood.2019003715 32202637 PMC7332900

[82] Li R Tang H Burns JC Hopkins BT Le Coz C Zhang B. BTK inhibition limits B-cell-T-cell interaction through modulation of B-cell metabolism: implications for multiple sclerosis therapy. Acta Neuropathol (2022) 143(4):505–21. doi: 10.1007/s00401-022-02411-w PMC8960592 35303161

[83] Chen GM Melenhorst JJ Tan K. B cell targeting in CAR T cell therapy: Side effect or driver of CAR T cell function? Sci Transl Med (2022) 14(650):eabn3353. doi: 10.1126/scitranslmed.abn3353 35731887 PMC9284935

[84] Zanatta E Cozzi M Marson P Cozzi F. The role of plasma exchange in the management of autoimmune disorders. Br J Haematol (2019) 186(2):207–19. doi: 10.1111/bjh.15903 30924130

[85] Hoffmann A Kerr S Jellusova J Zhang J Weisel F Wellmann U. Siglec-G is a B1 cell-inhibitory receptor that controls expansion and calcium signaling of the B1 cell population. Nat Immunol (2007) 8(7):695–704. doi: 10.1038/ni1480 17572677

[86] Tedder TF. B10 cells: a functionally defined regulatory B cell subset. J Immunol (2015) 194(4):1395–401. doi: 10.4049/jimmunol.1401329 25663677

[87] Cardinal H Dieudé M Brassard N Qi S Patey N Soulez M. Antiperlecan antibodies are novel accelerators of immune-mediated vascular injury. Am J Transplant (2013) 13(4):861–74. doi: 10.1111/ajt.12168 23432943

[88] Bröker K Figge J Magnusen AF Manz RA Köhl J Karsten CM. A novel role for C5a in B-1 cell homeostasis. Front Immunol (2018) 9:258. doi: 10.3389/fimmu.2018.00258 29520270 PMC5827565

[89] Lee KM Kim JI Stott R Soohoo J O'connor MR Yeh H. Anti-CD45RB/anti-TIM-1-induced tolerance requires regulatory B cells. Am J Transplant (2012) 12(8):2072–8. doi: 10.1111/j.1600-6143.2012.04055.x PMC3396747 22494812

[90] Deng S Moore DJ Huang X Lian MM Mohiuddin M Velededeoglu E. Cutting edge: transplant tolerance induced by anti-CD45RB requires B lymphocytes. J Immunol (2007) 178(10):6028–32. doi: 10.4049/jimmunol.178.10.6028 17475825

[91] Hernandez A Patil NK Brewer M Delgado R Himmel L Lopez LN. Pretreatment with a novel Toll-like receptor 4 agonist attenuates renal ischemia-reperfusion injury. Am J Physiol Renal Physiol (2023) 324(5):F472–f482. doi: 10.1152/ajprenal.00248.2022 36995924 PMC10151043

[92] Huang E Vo A Choi J Ammerman N Lim K Sethi S. Three-year outcomes of a randomized, double-blind, placebo-controlled study assessing safety and efficacy of C1 esterase inhibitor for prevention of delayed graft function in deceased donor kidney transplant recipients. Clin J Am Soc Nephrol (2020) 15(1):109–16. doi: 10.2215/cjn.04840419 PMC6946080 31843975

[93] Kaabak M Babenko N Shapiro R Zokoyev A Dymova O Kim E. A prospective randomized, controlled trial of eculizumab to prevent ischemia-reperfusion injury in pediatric kidney transplantation. Pediatr Transplant (2018) 22(2):e13129. doi: 10.1111/petr.13129 29377474

